# Supplementary material for: Do people with disabilities experience disparities in cancer care? A systematic review
Source: PLoS One. 2023 Dec 13;18(12):e0285146. doi: 10.1371/journal.pone.0285146 (PMC10718463; doi:10.1371/journal.pone.0285146)
Supplement: Attachment — Submitted filename: Comments+PP.docx [file pone.0285146.s002.docx]

This systematic review aimed to bridge an existing gap in the literature by conducting a comprehensive analysis of studies spanning a period of over two decades, addressing issues ranging from inadequate training of healthcare workers to the necessity for more inclusive clinical trials. Nevertheless, there are minor aspects of the study that necessitate additional clarification and improvement before considering the manuscript for publication. I have outlined these points in the comments provided below:

**1. Abstract:** The abstract provides a concise summary of the systematic review article. It effectively presents the background, methods, results, and discussion. However, I suggest including more specific details on the inclusion and exclusion criteria applied in the systematic review to enhance transparency and replicability. Additionally, it would be beneficial to briefly discuss the implications of the findings and potential strategies for addressing the identified disparities in cancer care for people with disabilities.

**2. Introduction:** The introduction provides a broad overview of the topic, highlighting the burden of cancer, the importance of accurate diagnosis and access to appropriate treatment, and the disparities faced by people with disabilities. I suggest providing some statistics to support the statements regarding the disparities in the use of cancer prevention services and lower cancer screening rates as factors contributing to higher mortality rates in people with disabilities. Also, consider emphasizing the potential impact of addressing healthcare disparities for people with disabilities on overall health outcomes and mortality rates.

**3. Methods:** The methods section provides a detailed systematic review process. However, consider discussing any efforts made to minimize publication bias. Additionally, it would be beneficial for the authors to provide a brief rationale for excluding non-English studies. Regarding the risk of bias assessment, report how many reviewers assessed the risk of bias in each study.

**4. Results:** The results section provides a comprehensive overview of the findings from the systematic review. It covers the study selection process, characteristics of included studies, types of disabilities and cancer, outcomes assessed, risk of bias assessment, and specific results for each outcome. It would be helpful to discuss if there were any patterns or differences in outcomes among the different types of disabilities or cancers. Are there certain subgroups within disabilities or cancer types that experience more significant disparities in cancer care? What about the clinical significance of the findings? How do these disparities in cancer care for people with disabilities impact patient outcomes, quality of life, and healthcare delivery?

**5. Discussion:** I suggest that the authors explicitly mention the gaps in knowledge or research questions that remain unanswered based on the findings of this study. Also, consider discussing strategies for promoting the inclusion of people with disabilities in clinical trials and highlight the challenges that exist in communication and partnership between healthcare providers and individuals with disabilities.

Overall, this systematic review article appears to be well-structured, with a comprehensive search strategy, a clear presentation of results, and a thoughtful discussion of limitations. The findings have significant implications for healthcare policies and practices, particularly in addressing the disparities in cancer care for individuals with disabilities. I would recommend that the authors continue with the manuscript submission process while addressing the minor comments and suggestions for improvement mentioned above.
