# Supplementary material for: Do people with disabilities experience disparities in cancer care? A systematic review
Source: PLoS One. 2023 Dec 13;18(12):e0285146. doi: 10.1371/journal.pone.0285146 (PMC10718463; doi:10.1371/journal.pone.0285146)
Supplement: Attachment — Submitted filename: Response to Reviewers.docx [file pone.0285146.s004.docx]

**Response to Reviewers**	November 11th, 2023

Manuscript title: Disparities in cancer care for people with disabilities: a systematic literature review.

Dr. Sina Azadnajafabad, MD, MPH

Academic Editor

PLOS ONE

*Dear Dr. Azadnajafabad,*

*Thank you for inviting us to submit a revised draft of our manuscript entitled, " Do people with disabilities experience disparities in cancer care? A systematic review" to PLOS ONE. We also appreciate the time and effort you and each of the reviewers have dedicated to providing insightful feedback on ways to strengthen our paper. Thus, it is with great pleasure that we resubmit our article for further consideration. We apologize for the long delay in completing this second revision, but unfortunately one of the Authors was absent for several weeks due to sudden health problems. We hope that the responses we provide below satisfactorily address all the issues and concerns that you have noted.*

*To facilitate your review of our revisions, the following is a point-by-point response to the questions and comments delivered in your letter dated August 24th, 2023.*

Additional Editor Comments:

The manuscript needs edits regarding the manuscript formatting and sections of the paper according to the Plos One requirements.

*Thank you; every section has been now revised according to Plos One requirements.*

Also, the referencing format is not according to the journal style, besides the errors evident in the number of cited manuscript which need a major revision.

*The referencing format has been changed to the one required by Plos One guidelines, and the numbers of cited manuscripts have been corrected and listed in the proper order.*

*Thank you again, Dr. Azadnajafabad and Reviewers, for giving us the opportunity to strengthen our manuscript with your valuable comments and queries We have worked to incorporate your feedback and hope that these revisions persuade you to accept our submission.*

*Sincerely,*

*Dr. Irene Tosetti, MD, MBA, MPH*

*Corresponding Author*
